# Research trends on the gut microbiota in endocrine metabolism: a thematic and bibliometric analysis

**DOI:** 10.3389/fcimb.2024.1371727

**Published:** 2024-03-22

**Authors:** Durmus Doğan, Taylan Çelik

**Affiliations:** ^1^ Department of Pediatric Medicine, Division of Pediatric Endocrinology, Çanakkale Onsekiz Mart University, Çanakkale, Türkiye; ^2^ Department of Pediatric Medicine, Division of Pediatric Infectious Diseases, Çanakkale Onsekiz Mart University, Çanakkale, Türkiye

**Keywords:** gut microbiota, bibliometric analysis, endocrinology, probiotics, short-chain fatty acid

## Abstract

**Background:**

Gut microbiota studies in the field of endocrinology metabolism have attracted increasing attention in recent years. To comprehensively assess the evolving landscape of this research field, we conducted a thorough bibliometric analysis of gut microbiota studies in endocrinology metabolism indexed in the Web of Science database.

**Methods:**

We collected and analyzed 3,339 original research articles and reviews published from 1972 to 2023. Using various bibliometric indicators, we investigated publication trends, country contributions, international collaborations, prolific authors, top journals, and influential articles.

**Results:**

Our analysis revealed a significant upsurge in publications after 2010, indicating a growing scientific interest in microbiota and endocrinology metabolism. Keyword and thematic analyses have identified gut microbiota, obesity, diabetes, and inflammation as core research themes. Additionally, the roles of probiotics and prebiotics are increasingly researched for their therapeutic effects in shaping the microbiota.

**Conclusion:**

This study reveals that research in endocrinology metabolism is increasingly decoding the connection between gut microbiota and diseases. There’s also a growing focus on microbiota manipulation, which points to a shift towards personalized medicine. Future research should focus on integrating these findings into clinical practice, moving from lab-based studies to real-world patient care.

## 1 Introduction

Gut commensals, as the largest symbiotic microbiota, are indispensable for the human body. It has been estimated that the microbes in our bodies collectively make up to 100 trillion cells, tenfold the number of human cells (Qin et al., 2010). Recent evidence strongly suggests that these microorganisms function almost like an additional organ, actively participating in shaping and maintaining our physiology (Qi et al., 2021). The gut microbiota plays a pivotal role in regulating hormone levels, responding to host hormones, and even producing its hormones (Sudo, 2014). As a result, it is considered a fully-fledged endocrine organ, exerting a range of effects that extend to distant organs and pathways (Qi et al., 2021). The intricate relationship between microbiota and hormones carries profound implications for various aspects of health, behavior, metabolism, immunity, and reproduction (Neuman et al., 2015).

Healthy gut microbiota is composed of 6 phyla including *Firmicutes, Bacteroidetes, Actinobacteria, Proteobacteria, Fusobacteria*, and *Verrucomicrobia* (Crudele et al., 2023; Hamjane et al., 2024). The two phyla *Firmicutes* and *Bacteroidetes* represent 90% of the intestinal microbiota (Hamjane et al., 2024). Changes in the composition of the microbiota can significantly impact health. These changes can be evaluated in the context of cause or consequence. However, it is undeniable that today, the gut microbiota works in synergy with our body’s systems to influence health profoundly.

The interaction between microbiota and hormones is bidirectional. As demonstrated in William’s review, hormones possess the capacity to directly impact the diversity and composition of the microbiota, while conversely, the microbiota can modulate hormone production and mediate hormonal functions (Williams et al., 2020). The composition of the gut microbiota varies by sex hormones, dysregulation of the Hypothalamic-Pituitary-Adrenal (HPA) axis and insulin, feeding behavior, and obesity (Yoon and Kim, 2021; Farzi et al., 2018; Kelly et al., 2015; Rusch et al., 2023; He et al., 2021). The gut microbiota plays a pivotal role in regulating feeding behaviors and metabolism by interacting with hormones like insulin, ghrelin, and GLP-1 (Williams et al., 2020). Studies investigating the relationship between gut microbiota and obesity explain that gut microbiota can modify host metabolism and the role of dysbiotic gut microbiota in the development of obesity (Qi et al., 2021; Angelakis et al., 2012; Everard et al., 2013). The dozens of metabolites produced by gut microbiota impact energy regulation and insulin sensitivity (Qi et al., 2021; Wahlström et al., 2016). Metabolites such as short-chain fatty acids (SCFA) and bile acids play an important role in central pathologies of metabolic syndrome such as insulin resistance; these metabolites are products of the gut microbiota that affect energy balance and insulin sensitivity (Wahlström et al., 2016; Den Besten et al., 2015). Additionally, anti-diabetic medicines positively impacted levels of butyrate and propionate by promoting microbiota growth responsible for SCFA production. Understanding the various effects of intestinal bacterial metabolites in the development of endocrine disease is becoming critical for the discovery of new targets against metabolic diseases and the development of new drugs. The potential for these microbiota-driven effects to inform innovative treatments is profound, necessitating further investigation into their foundations.

Bibliometrics, a library and information science branch, is concerned with the quantitative analysis of bibliometric data. It primarily focuses on academic productivity, and it analyzes the bibliometric data of published articles, books, and conference papers in a specific field. Bibliometric analysis deals with the quantitative analysis of citations, citation counts, the significance of a research topic, and research conducted in certain geographic regions. The data examined by bibliometric analysis typically involves large quantities of objective data. These analyses provide a comprehensive view of the field’s evolution, collaboration trends, and knowledge dissemination that are essential for understanding the global impact and direction of this research area. Recently, bibliometric study and visualization analysis have become indispensable methods for literature analysis in the field of medical research (Ellegaard and Wallin, 2015; Guler et al., 2016).

The analysis herein is crafted to address the noted scarcity of comprehensive bibliometric analyses that encompass the entire spectrum of endocrinology and metabolism research. While there exist segmented bibliometric studies focusing on specific intersections within the field-such as the gut microbiota’s role in diabetes, the impact on bone health, and the interplay with obesity-our research intends to bridge the gap by delivering a holistic overview of the research landscape (Zhang et al., 2022; Zhang et al., 2023; Su et al., 2023).

By examining the field as a whole, the study will identify critical research areas, highlight existing gaps, and underline emerging trends. The resulting insights can facilitate the development of informed strategies for research funding, academic focus, and policy making. Furthermore, this analysis aims to uncover collaborative models and network structures that foster interdisciplinary and international partnerships, which are crucial for advancing the frontiers of endocrinology and metabolism research.

## 2 Materials and methods

### 2.1 Introduction of database

In our bibliometric analysis, we utilized the Web of Science Core Collection (WoSCC) as the primary data source. To acquire terms associated with the gastrointestinal microbiota, we conducted searches in PubMed’s Medical Subject Headings (MeSH) using keywords such as “gastrointestinal microbiomes,” “probiotics,” “prebiotics,” and “synbiotics.” These obtained terms were subsequently employed in our search within the WoSCC database, following the search strategy outlined as follows: ‘‘Topic: [(Gastrointestinal Microbiome OR Gastrointestinal Microbiomes OR Microbiome, Gastrointestinal OR gut microbiota OR gut microbiotas OR Microbiome, Gut OR Gut Microflora OR Microflora, Gut OR Gut Microbiota OR Gut Microbiotas OR Microbiota, Gut OR Gastrogut microbiota OR Flora, Gastrointestinal OR gut microbiota OR Flora, Gut OR Gastrointestinal Microbiota OR Gastrointestinal Microbiotas OR Microbiota, Gastrointestinal OR Gastrointestinal Microbial Community OR Gastrointestinal Microbial Communities OR Microbial Community, Gastrointestinal OR Gastrointestinal Microflora OR Microflora, Gastrointestinal OR Gastric Microbiome OR Gastric Microbiomes OR Microbiome, Gastric OR Intestinal Microbiome OR Intestinal Microbiomes OR Microbiome, Intestinal OR Intestinal Microbiota OR Intestinal Microbiotas OR Microbiota, Intestinal OR Intestinal Microflora OR Microflora, Intestinal OR gut microbiota OR Flora, Intestinal OR Enteric Bacteria OR Bacteria, Enteric OR dysbiosis OR probiotics OR prebiotics OR synbiotics)].

We then refined our search within WoSCC by applying the following criteria:

- Category Selection: We selected the “Endocrinology Metabolism” category to focus on articles most relevant to our research on the intersection of endocrine function and metabolic processes.
- Document Type: We included various types of documents, such as research articles, in our analysis to obtain a comprehensive view of the field.
- Language Criteria: We limited our search to English-language publications.
- Indexing Databases: We narrowed down our search to the Science Citation Index Expanded (SCIE) and Emerging Sources Citation Index (ESCI) to ensure that we were capturing the most pertinent and impactful studies.
- Exclusion Criteria:
- Non-peer-reviewed materials, such as editorials and conference abstracts, were excluded to maintain the scientific rigor of our analysis.

**Figure 1:**
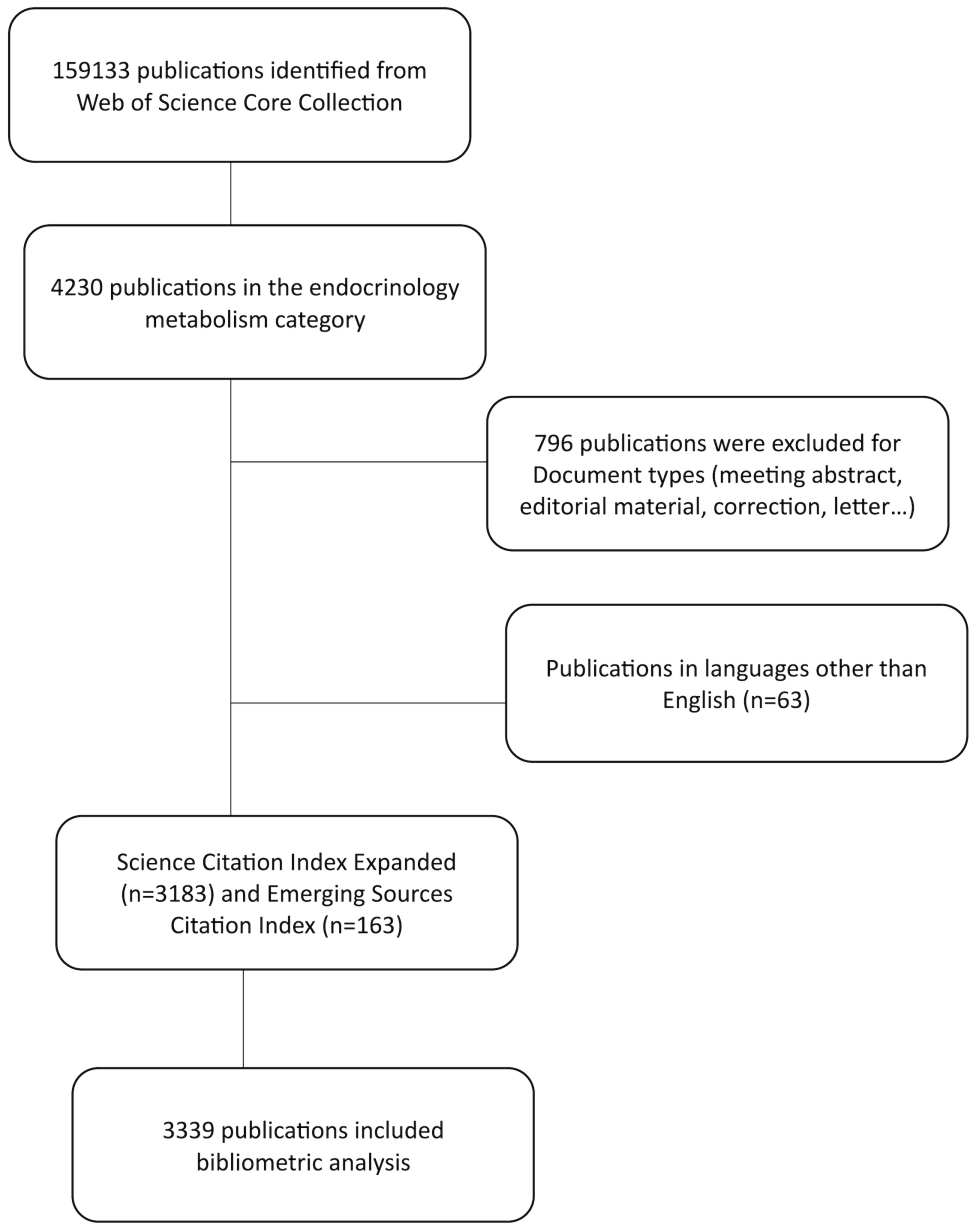
Flow diagram of the literature selection process.

The initial search resulted in identifying 159133 publications. After applying the inclusion and exclusion criteria as outlined above and depicted in **Figure 1**, we narrowed our focus to a final dataset of 3339 publications, which were included in the bibliometric analysis. The “Full Record and Cited References” of the selected publications were then exported in “Plain Text” and “Tab Delimited file” formats for subsequent analysis.

### 2.2 Data analysis and visualization

R studio 4.3.1 (Aria and Cuccurullo, 2017) and VOSviewer 1.6.18 (van Eck and Waltman, 2010) were utilized for data analysis and visualization. The open-source software packages Bibliometrix and Biblioshiny running in the R language environment were also utilized to obtain key bibliometric analyses such as most productive authors, journals, institutions, countries, most cited documents, and keyword analysis (Aria and Cuccurullo, 2017). VOSviewer was applied to visualize co-authorships of authors; collaboration of countries, and institutions; bibliographic coupling of citations; co-occurrence of all keywords, and related knowledge maps.

Keyword analysis is a predominant component of academic research, playing a pivotal role in summarizing study topics within specific fields and exploring current trends and research frontiers (Li J. et al., 2023). Within the framework of WoS records, two distinct types of keywords are employed: Author Keywords, thoughtfully chosen by the original authors to best encapsulate their articles’ content, and Keywords Plus, automatically generated by a computer algorithm based on recurring terms found in the titles of an article’s references. These terms may not necessarily align with the article’s title or Author Keywords (Zhang et al., 2016; Garfield and Sher, 1993). As argued by Garfield, Keywords Plus terms can capture an article’s content with greater depth and a wider range (Garfield and Sher, 1993). Consequently, Keywords Plus terms serve as instrumental tools for describing and tracking research trends across various scientific fields. By embracing keywords and Keywords Plus terms, researchers can effectively navigate the ever-evolving landscape of academic literature and remain abreast of the latest developments in their respective areas of study.

Synonyms of keywords were systematically analyzed, and synonyms were merged for more accurate results. Files were created in “thesaurus_terms.txt” format for VOSviewer and “synonym_text.csv” format for Bibliometrix. In these files, terms such as intestinal microbiome, gastrointestinal microflora, gut microbiome, etc., were combined under the name gut microbiota. Additionally, the term microbiome was merged with microbiota and accepted as different from the word gut microbiota. This methodical approach to synonym integration, which included grouping words with overlapping semantic fields, aimed to enhance the precision of word analysis within our bibliometric study.

### 2.3 Application of Bradford’s law and h-index in journal categorization

In our bibliometric analysis, we employed Bradford’s Law to categorize journals, pinpointing a core collection that contributes the majority of articles on endocrinology metabolism. This approach allowed us to arrange journals from those with the most publications to those with the fewest, thereby forming Zone 1 which consists of the journal cluster responsible for one-third of all publications. Zone 2 includes journals with moderate publication frequency, while Zone 3 comprises those with fewer articles. Bradford’s Law particularly assists in identifying “core sources” and serves as a crucial tool in distinguishing the most significant journals within a research field.

In addition to utilizing Bradford’s Law for journal categorization, we incorporated the h-index to evaluate the impact of journals within the specific scope of endocrinology and metabolism. The h-index is commonly understood as a metric that quantifies the productivity of a journal based on the number of publications and their citation counts, and it serves as a reliable indicator of a journal’s academic influence. For the purposes of our study, the h-index was calculated based on the number of articles in the field of endocrinology and metabolism that have been cited at least ‘h’ times, thus providing a domain-specific measure of impact. This value, therefore, differs from the overall h-index of a journal, which encompasses all subject areas it covers. By applying both Bradford’s Law and these field-specific h-index calculations, we were able to identify and rank the journals that are most influential in endocrinology and metabolism research.

### 2.4 Thematic map and evaluation

We conducted a thematic evaluation that encompasses the changes and trends in endocrinology and metabolism research. Initially, the period from 2010 to 2023 was divided into two equal time frames for thematic evaluation and visualization. The thematic map was created using keywords from the authors’ publications. The current thematic map evaluates the last six years. This map illustrates how the focus of research has shifted and which areas have gained or lost importance. In this map, themes are categorized into four groups: Niche themes are specialized topics that are not widely researched but are crucial for advancements in specific areas of endocrinology and metabolism. They represent emerging fields or highly specialized interests within the area. Motor themes are dynamic themes that drive the research field forward. They often indicate areas with a substantial current interest in research. Basic themes have a long history of research and represent established knowledge that supports ongoing studies and progress. Emerging/Declining themes indicate research areas that are either increasing or decreasing in popularity and relevance (Cobo et al., 2011; Aria et al., 2022).

## 3 Results

### 3.1 Annual growth trend

**Figure 2:**
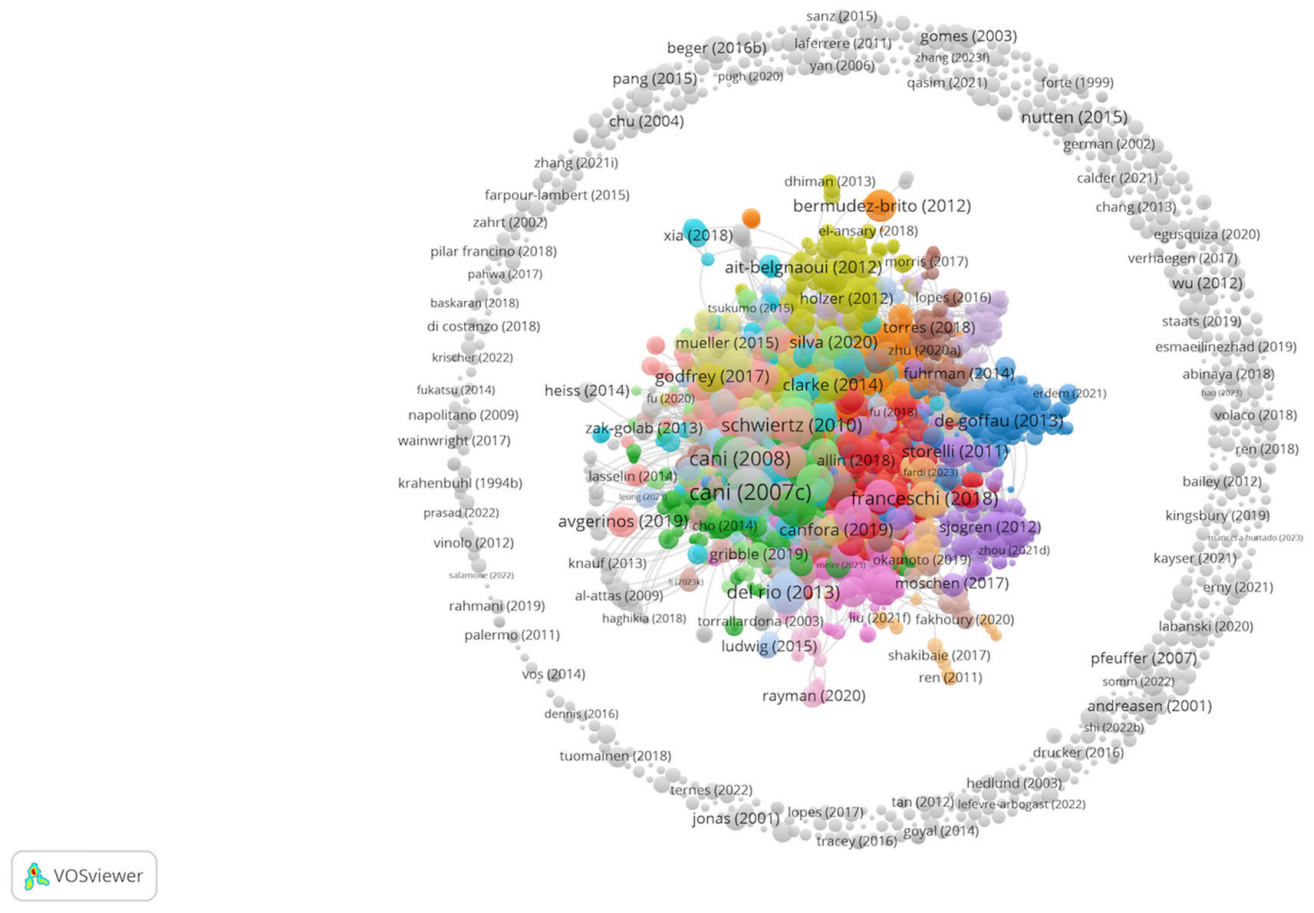
Network Visualization of gut microbiota publications to explore from the WoS Database in Endocrinology Metabolism categories. The network visualization represents 3339 publications retrieved from the WoS database with at least 0 citations using the VOSviewer program. All articles are included, and their nodes are weighted based on the number of citations. The visualization parameters include a resolution of 0.3, maximum lines (10,000), and normalization of associated strengths. Central clusters indicate highly cited research, while peripheral nodes show less cited works, demonstrating the field’s citation landscape.

**Figure 3:**
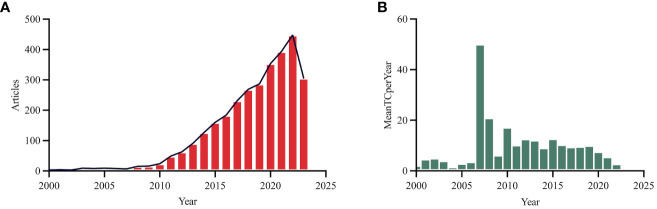
**(A)** Annual scientific production between 2000-2023. **(B)** Avarage citaion per year. MeanTCperYear: Meantime citation per year.

In the category of gastrointestinal microbiota and endocrinology metabolism, the WoSCC database encompasses a total of 3,339 original research articles and reviews, contributed by 15,861 authors from 178 sources, covering the period from 1972 to 2023 as of August 25, 2023. These publications feature a significant level of international collaboration, accounting for 26.71% of total articles (**Table 1**). The dataset comprised 144,246 references, resulting in an average of 51.09 citations per article. **Figure 2** offers a macroscopic overview of this dataset displaying clusters of highly cited (as seen central cluster) articles as well as less cited (as seen peripheral ring) articles in this field. This visualization is structured with nodes representing individual articles, weighted proportionally by their citation counts to reflect their impact within the domain. The central, densely interconnected nodes highlight influential articles foundational to gut microbiota research. Complementing this, **Figure 3** presents two distinct charts reflecting the annual scholarly output and its citation impact. The first chart details the growth in publication volume over time, revealing a surge post-2010. The annual growth rate averaged 11.9%, with a notable increase of 26.8% between 2000 and 2023. The earliest recorded study related to gut microbiota in the realm of endocrinology and metabolism dates to 1972. However, only 33 publications met the search criteria and were included in the dataset until the year 2000. The second chart of **Figure 3** provides a year-wise average citation count per article, with 2007 and 2008 standing out for higher rates.

**Table 1:** Main information about bibliometric analysis data.

Item	Results
Timespan	1972- 2023
Sources (Journals, Books, etc)	178
Documents	3339
Annual Growth Rate (%)	11.09
Document Average Age	4.91
Average citations per doc	51.09
References	144246
Keywords Plus	6450
Author Keyword	5215
Authors	15861
Single-authored docs	161
Co-Authors per Doc	6.72
International co-authorships (%)	26.71
Corresponding Countries	69
Institution	180
Document Types	
Article	2156
Review	1183

### 3.2 Analysis of countries and collaboration

A total of 94 countries contributed to the 3,339 publications, of which 71 were the corresponding author’s countries. The United States (n=680, 20.4%) emerged as the leading producer of publications, closely followed by China (n=658, 19.7%), Italy (n=190, 5.7%), France (n=146, 4.4%), and Canada (n=146, 4.4%) as illustrated in **Table 2**. Notably, the United States secured the highest number of citations with 37,166 citations, followed by France with 17,004 citations, and China with 14,536 citations. Among the top 20 countries, Sweden had the highest average citations per publication (140.1), followed by France (116.5) and Belgium (108.1). The average number of citations per publication for the United States is 54.7 and for China 22.1. Among the 20 countries that produced the highest number of articles, the Netherlands, Denmark, and Sweden had 53.8%, 50.7%, and 50% international links, respectively, and were found to have more international collaborations than the United States and China (**Table 2**).

**Table 2:** Top 20 countries by number of publications and citations.

Corresponding author’s countries	Publications	MCP_ratio	Corresponding author’s countries	Total citations	Average article citations
United States	680	0.224	United States	37166	54.70
China	658	0.15	France	17004	116.50
Italy	190	0.326	China	14536	22.10
Canada	146	0.253	United Kingdom	13005	98.50
France	146	0.315	Sweden	9149	48.20
United Kingdom	132	0.386	Italy	8685	140.10
Spain	105	0.21	Netherlands	8094	87.00
Iran	104	0.192	Canada	7101	48.60
Netherlands	93	0.538	Belgium	6926	94.90
Japan	85	0.094	Spain	5836	108.10
Australia	80	0.313	Germany	5352	51.00
Germany	76	0.434	Denmark	4832	63.60
Brazil	74	0.203	Brazil	3838	51.90
Denmark	73	0.507	Australia	3604	45.00
India	64	0.266	Finland	3217	59.60
Sweden	62	0.5	Ireland	2731	70.00
Belgium	54	0.463	Iran	2339	58.50
Finland	54	0.444	Switzerland	2331	27.40
Korea	48	0.188	Japan	2278	21.90
Switzerland	40	0.4	Austria	1517	66.00

MCP, Multiple Country Publications; MCP_ratio, The percentage of publications that have resulted from international collaborations.

### 3.3 Source analysis

**Figure 4:**
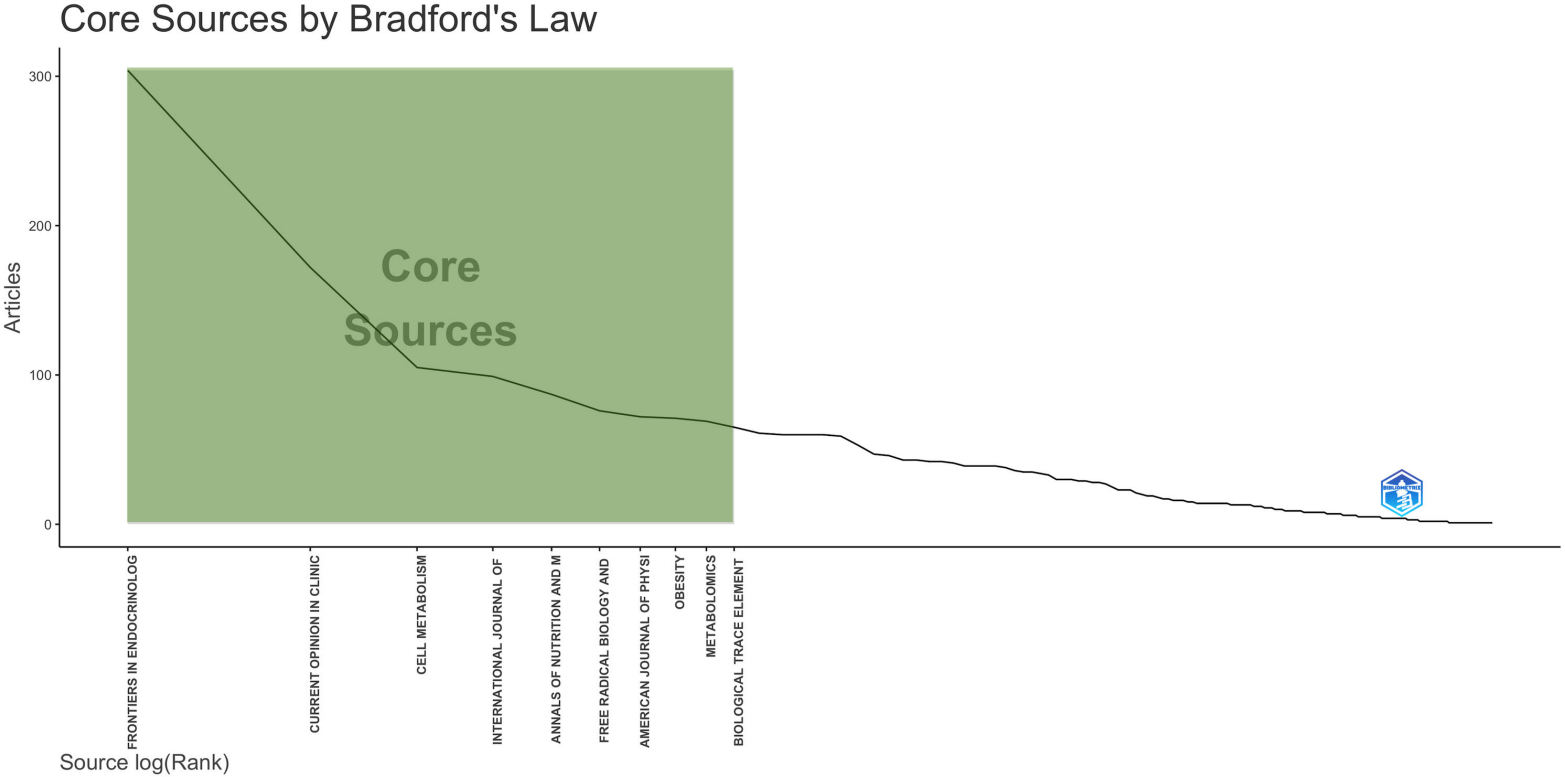
Bradford**’**s Law.

When analyzing the most relevant sources, *Frontiers in Endocrinology* emerged as the top publisher with a total of 304 articles. We also employed Bradford’s Law zones to identify the main journals in this category (**Figure 4**). According to Bradford’s Law, Zone 1 (Core) included 10 journals with a total of 1,120 publications between 2000 and 2023. The top 5 journals in this category were *Frontiers in Endocrinology*, *Current Opinion in Clinical Nutrition and Metabolic Care*, *Cell Metabolism*, *International Journal of Obesity*, and *Annals of Nutrition and Metabolism*.

Furthermore, in our analysis of h-index rankings, which gauge journal impact through citation counts, it’s noteworthy that both *Diabetes* and *Diabetologia*, located within Zone 2, attained among the top 10 journals with the highest h-index values. **Table 3** displays the 25 most influential journals based on their h-index values (**Table 3**). The top three journals according to the h-index ranking were *Cell Metabolism, Current Opinion in Clinical Nutrition and Metabolic Care, and International Journal of Obesity*. In the **Supplementary Material**, a visualization of the bibliographic coupling network of these sources is presented, with nodes representing journals and their sizes, showing citation weight (**Supplementary Material**).

**Table 3:** Ranking of the 25 journals with the highest local impact, assessed by h-index.

Journals	Zone	h_index	TC	NP	PY_start
Cell Metabolism	Zone 1	71	21571	105	2011
Current Opinion in Clinical Nutrition and Metabolic Care	Zone 1	43	6504	172	2001
International Journal of Obesity	Zone 1	41	7943	99	1988
Diabetes	Zone 2	39	16026	61	2007
Diabetologia	Zone 2	36	5875	60	2005
Frontiers in Endocrinology	Zone 1	35	5010	304	2014
Free Radical Biology and Medicine	Zone 1	33	4518	76	2001
Annals Of Nutrition and Metabolism	Zone 1	31	4397	87	1998
Journal Of Clinical Endocrinology & Metabolism	Zone 1	31	5266	71	2008
Obesity	Zone 1	30	2929	60	1994
American Journal of Physiology-Endocrinology Metabolism	Zone 1	28	2401	72	1996
Metabolism-Clinical and Experimental	Zone 2	28	4619	43	1977
Molecular Metabolism	Zone 2	28	2757	59	2012
Trends In Endocrinology and Metabolism	Zone 2	27	3811	39	2004
Diabetes Care	Zone 2	27	3441	46	2011
Endocrinology	Zone 2	24	2334	42	2010
Obesity Reviews	Zone 2	24	2790	47	2011
Current Diabetes Reports	Zone 2	23	1475	43	2010
Nature Reviews Endocrinology	Zone 2	22	7467	27	2010
Bifactors	Zone 2	21	1263	41	1991
Journal Of Diabetes Research	Zone 2	21	1315	39	2005
Journal Of Endocrinology	Zone 2	20	1245	42	2013
Metabolomics	Zone 1	20	1831	69	2007
Nutrition Metabolism and Cardiovascular Diseases	Zone 2	20	1630	39	2006
Psychoneuroendocrinology	Zone 2	20	2967	38	2012

Zone, Bradford’s law zones; TC, Time Cited; NP, Number of Publications; PY_start, Publication year start.

### 3.4 Most active organizations and collaborations

**Figure 5:**
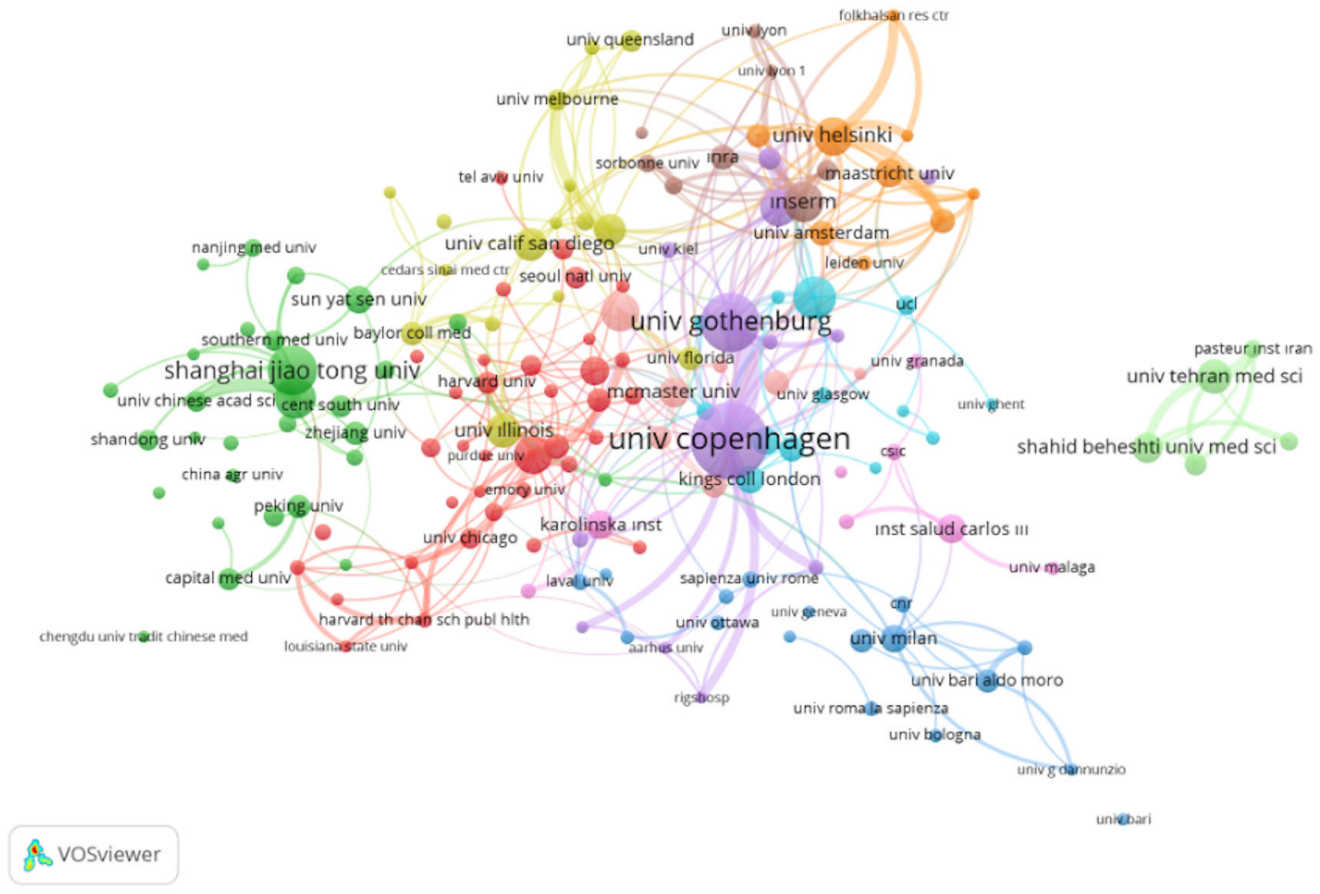
Visualization of the co-authorship network map of institutions where a minimum of 10 documents. Node size indicates total link strengths.

The University of Copenhagen stands out as the institution with the highest number of published papers (n= 175), followed by Shanghai Jiao Tong University (n= 97), the University of Gothenburg (n= 93), the Tehran University of Medical Sciences (n= 93), the University of Toronto (n= 89), and the University of Turku (n= 87). **Figure 5** displays the network of institutional collaborations in the research field. This map includes 162 institutions that have produced at least 10 publications. Notably, the most substantial collaboration appears to be between the University of Copenhagen and the University of Gothenburg, as indicated by the total link strength (**Figure 5**).

### 3.5 Most relevant authors

**Figure 6:**
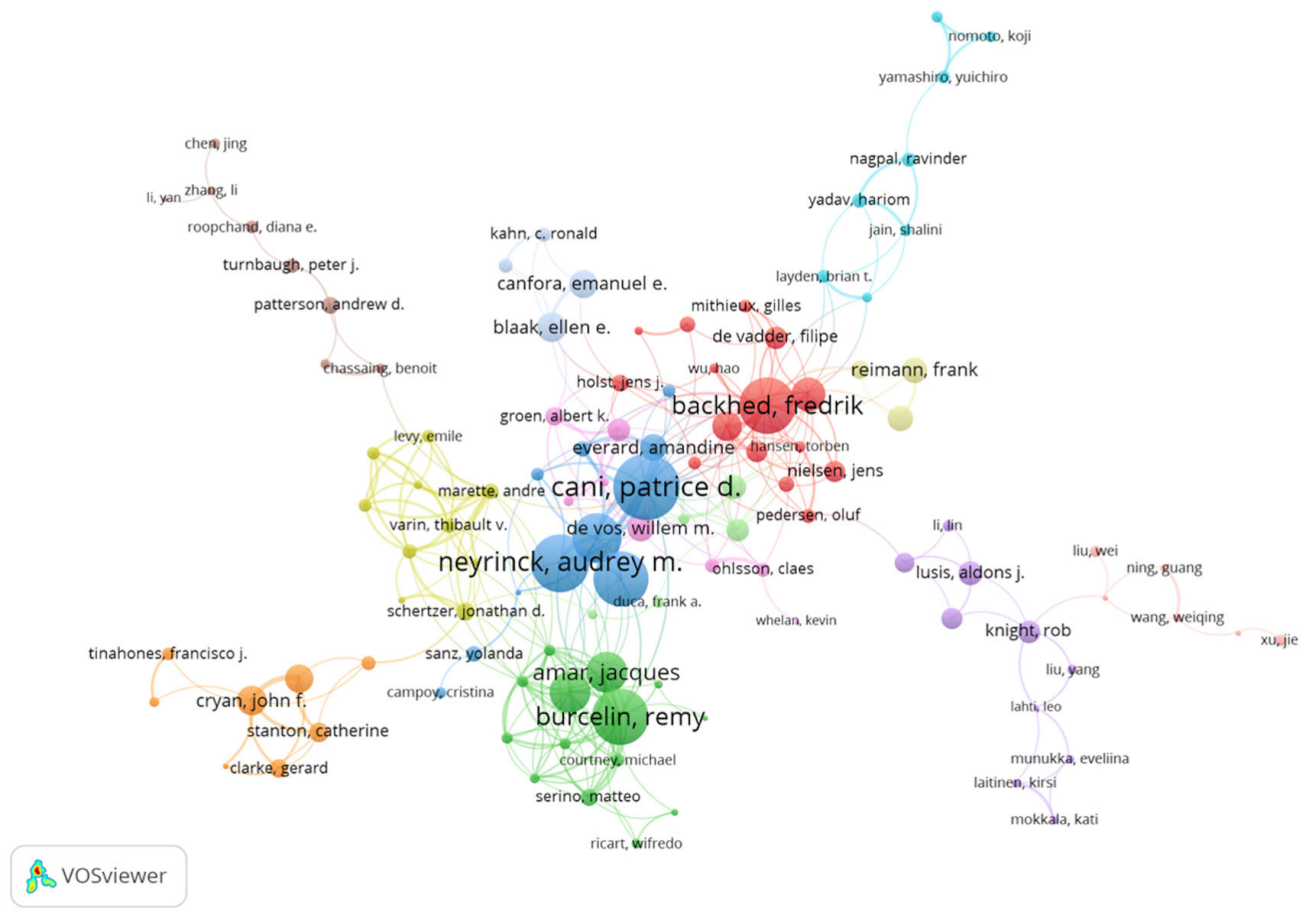
Co-Authorship network map of authors (large nodes indicate greater citation and the color of the cluster indicates collaborations).

**Table 4** displays the top 10 most productive authors in the field. These top 10 authors have written 243 articles. Cani PD emerges as the most prolific author with 39 articles. Other highly published authors include Bäckhed F and Nieuwdorp M. According to the h-index ranking, the three most active authors in this field are Bäckhed F, Cani PD, and Burcelin R (**Table 4**). **Figure 6** presents the co-authorship network map of authors.

**Table 4:** Top 10 authors by number of publications.

Authors	Articles	h- index	Citations counts
Cani PD	39	26	13326
Bäckhed F	35	27	9175
Nieuwdorp M	28	19	2603
Burcelin R	25	19	8922
Delzenne NM	25	19	11590
Cryan JF	21	17	2358
Schertzer JD	19	12	981
Marette A	17	12	746
Dipalma G	17	10	389
Clement K	16	11	1596

### 3.6 Document analysis

The most cited study, both globally and locally, is Cani PD et al. “Metabolic endotoxemia initiates obesity and insulin resistance” published in the *Diabetes* (Cani et al., 2007), followed by their work “Changes in gut microbiota control metabolic endotoxemia-induced inflammation in high-fat diet-induced obesity and diabetes in mice” published in the *Diabetes* (Cani et al., 2008). Subsequently, Del Rio D et al’s article, “Dietary (Poly)phenolics in Human Health: Structures, Bioavailability, and Evidence of Protective Effects Against Chronic Diseases” (Del Rio et al., 2013) published in 2013 in the *Journal of Antioxidants & Redox Signaling*, garnered significant attention.

**Table 5** presents the top 20 globally cited articles, with 12 of these articles amassing over 1000 citations each. The table also provides data on local citations and local citation rates for each article (**Table 5**).

**Table 5:** Top 20 most cited documents.

Rank	Author, year	Journal	Document	DOI	Local citations	Global citations	LC/GC Ratio (%)
1	Cani PD, (Cani et al., 2007)	Diabetes	Metabolic Endotoxemia Initiates Obesity and Insulin Resistance	10.2337/db06-1491	396	4158	9,52
2	Cani PD, (Cani et al., 2008)	Diabetes	Changes in Gut Microbiota Control Metabolic Endotoxemia-Induced Inflammation in High-Fat Diet–Induced Obesity and Diabetes in Mice	10.2337/db07-1403	312	3254	9,59
3	Del Rio D, (Del Rio et al., 2013)	Antioxidants & Redox Signaling	Dietary (Poly)phenolics in Human Health: Structures, Bioavailability, and Evidence of Protective Effects Against Chronic Diseases	10.1089/ars.2012.4581	7	1683	0,42
4	Schwiertz A, (Schwiertz et al., 2010)	Obesity	Microbiota and SCFA in Lean and Overweight Healthy Subjects	10.1038/oby.2009.167	170	1669	10,19
5	Buzzetti E, (Buzzetti et al., 2016)	Metabolism	The multiple-hit pathogenesis of non-alcoholic fatty liver disease (NAFLD)	10.1016/j.metabol.2015.12.012	14	1555	0,90
6	Sayin SI, (Sayin et al., 2013)	Cell Metabolism	Gut Microbiota Regulates Bile Acid Metabolism by Reducing the Levels of Tauro-beta-muricholic Acid, a Naturally Occurring FXR Antagonist	10.1016/j.cmet.2013.01.003	106	1398	7,58
7	Tolhurst G, (Tolhurst et al., 2012)	Diabetes	Short-Chain Fatty Acids Stimulate Glucagon-Like Peptide-1 Secretion via the G-Protein–Coupled Receptor FFAR2	10.2337/db11-1019	162	1361	11,90
8	Wahlstrom A, (Wahlström et al., 2016)	Cell Metabolism	Intestinal Crosstalk between Bile Acids and Microbiota and Its Impact on Host Metabolism	10.1016/j.cmet.2016.05.005	76	1353	5,62
9	Cani PD, (Cani et al., 2007)	Diabetologia	Selective increases of bifidobacteria in gut microflora improve high-fat-diet-induced diabetes in mice through a mechanism associated with endotoxaemia	10.1007/s00125-007-0791-0	138	1255	11,00
10	Canfora EE, (Canfora et al., 2015)	Nature Reviews Endocrinology	Short-chain fatty acids in control of body weight and insulin sensitivity	10.1038/nrendo.2015.128	85	1191	7,14
11	Franceschi C, (Franceschi et al., 2018)	Nature Reviews Endocrinology	Inflammaging: a new immune–metabolic viewpoint for age-related diseases	10.1038/s41574-018-0059-4	11	1186	0,93
12	Donohoe DR, (Donohoe et al., 2011)	Cell Metabolism	The Microbiome and Butyrate Regulate Energy Metabolism and Autophagy in the Mammalian Colon	10.1016/j.cmet.2011.02.018	62	1093	5,67
13	Kovatcheva-Datchary P, (Kovatcheva-Datchary et al., 2015)	Cell Metabolism	Dietary Fiber-Induced Improvement in Glucose Metabolism Is Associated with Increased Abundance of Prevotella	10.1016/j.cmet.2015.10.001	62	912	6,80
14	Furet JP, (Furet et al., 2010)	Diabetes	Differential Adaptation of Human Gut Microbiota to Bariatric Surgery–Induced Weight Loss: Links With Metabolic and Low-Grade Inflammation Markers	10.2337/db10-0253	137	860	15,93
15	Silva et al., 2020	Frontiers In Endocrinology	The Role of Short-Chain Fatty Acids From Gut Microbiota in Gut-Brain Communication	10.3389/fendo.2020.00025	22	855	2,57
16	Duncan SH, (Duncan et al., 2008)	International Journal Of Obesity	Human colonic microbiota associated with diet, obesity and weight loss	10.1038/ijo.2008.155	85	848	10,02
17	Everard A, (Everard et al., 2011)	Diabetes	Responses of Gut Microbiota and Glucose and Lipid Metabolism to Prebiotics in Genetic Obese and Diet-Induced Leptin-Resistant Mice	10.2337/db11-0227	86	760	11,32
18	Bennett BJ, (Bennett et al., 2013)	Cell Metabolism	Trimethylamine-N-Oxide, a Metabolite Associated with Atherosclerosis, Exhibits Complex Genetic and Dietary Regulation	10.1016/j.cmet.2012.12.011	41	672	6,10
19	Bermudez-Brito M, (Bermudez-Brito et al., 2012)	Annals of Nutrition and Metabolism	Probiotic Mechanisms of Action	10.1159/000342079	3	647	0,46
20	Clarke G, (Clarke et al., 2014)	Molecular Endocrinology	Minireview: Gut Microbiota: The Neglected Endocrine Organ	10.1210/me.2014-1108	41	644	6,37

### 3.7 Keywords and trend topics

**Figure 7:**
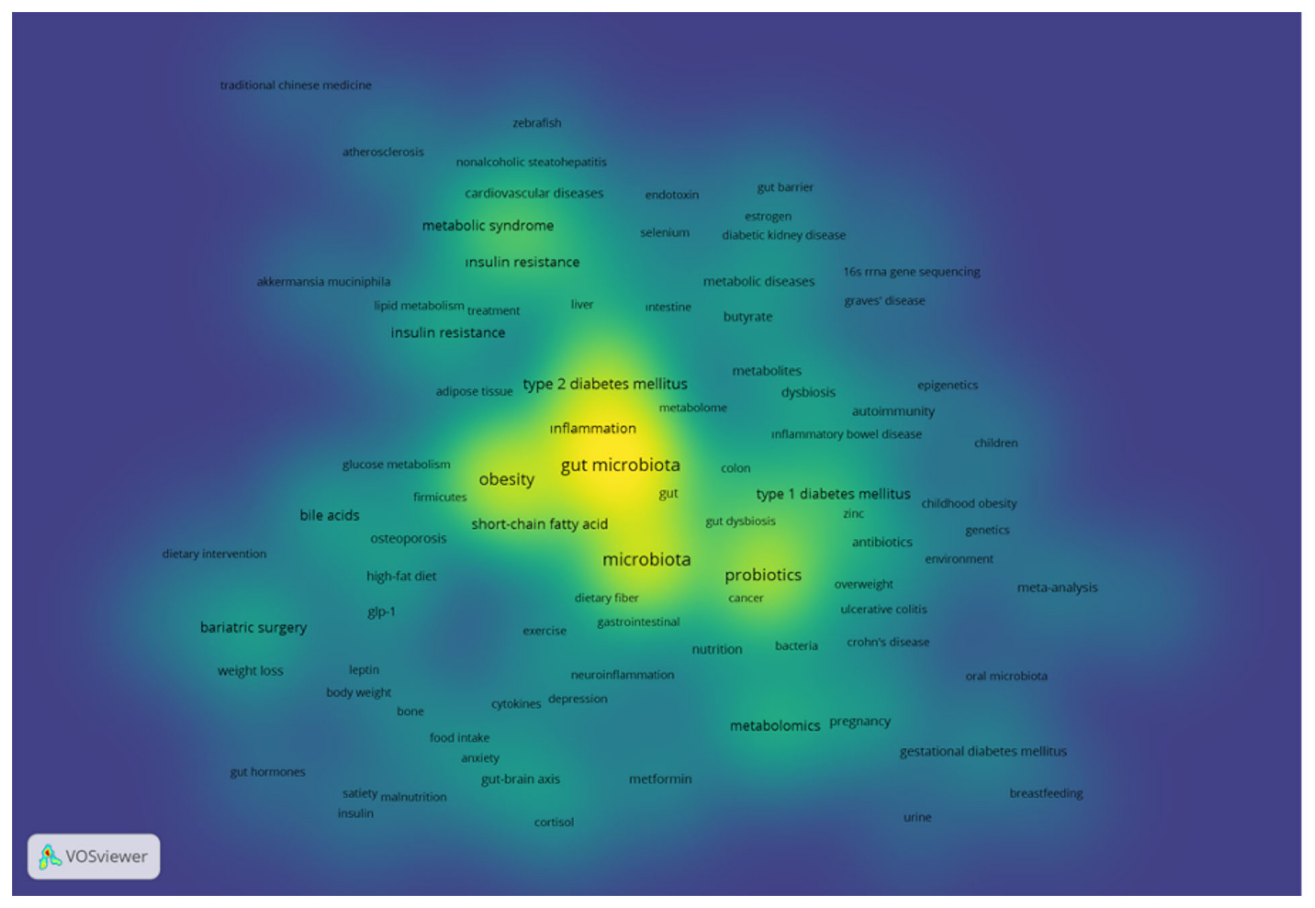
Density visualization of keyword co-occurrence network (the brighter color, the greater occurrence).

In this analysis, the 3339 articles included a total of 5215 Author Keywords and 6450 Keywords Plus. **Table 6** presents the top 20 Author Keywords and Keywords Plus terms with the highest frequency. Leading the list in terms of frequency is “gut microbiota” with 810 mentions, followed by “obesity” (n=521), “microbiota” (n=462), and “probiotic” (n=311). In the Keywords Plus category, the most frequently recurring terms are “gut microbiota” (n=1495), “obesity” (n=637), “insulin resistance” (n=374), and “inflammation” (n=342) (**Table 6**). **Figure 7** displays the density visualization of the co-occurrence network of the Author Keyword occurring 10 or more times (n=154) in the dataset.

**Table 6:** Most relevant author keywords and keywords plus.

Author Keywords	Frequency	Keywords Plus	Frequency
gut microbiota	810	gut microbiota	1495
obesity	521	obesity	637
microbiota	462	insulin-resistance	374
probiotic	311	inflammation	342
type 2 diabetes mellitus	233	chain fatty-acids	279
diabetes mellitus	221	metabolism	238
inflammation	214	glucagon-like peptide-1	197
insulin resistance	152	diet	194
Short-chain fatty acids	109	health	191
type 1 diabetes mellitus	100	expression	189
metabolic syndrome	87	microbiota	187
bariatric surgery	85	risk	181
metabolomics	85	mice	172
metabolism	81	disease	161
diet	72	oxidative stress	155
dysbiosis	72	weight-loss	154
prebiotics	69	association	148
oxidative stress	65	children	143
bile acids	55	insulin sensitivity	143
nutrition	54	adipose-tissue	141

**Figure 8:**
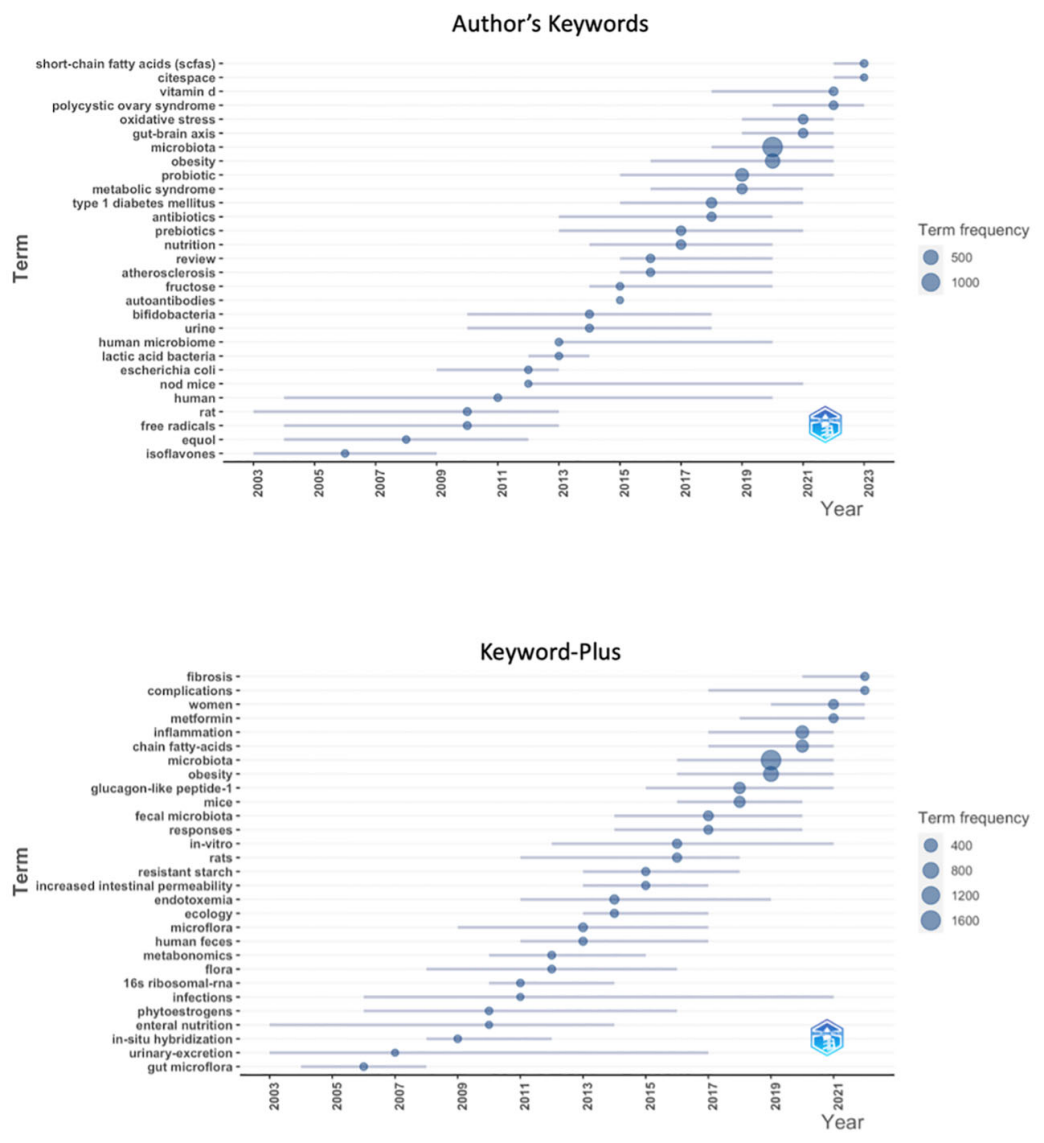
Trend topics of author keyword and keyword plus.

The keywords and Keywords Plus that define trends in a field change over time. For this reason, a trend topical analysis was conducted (**Figure 8**). At the beginning of 2000, the keywords preferred by the authors were “isoflavones”, “free radical”, “rat”, “*Escherichia coli”*, while in recent years, “short-chain fatty acids”, “CiteSpace”, “vitamin D”, “polycystic ovary syndrome”, and “gut-brain-axis”. In the Keywords Plus review, the trends topic changed from “gut microflora”, “urinary excretion”, “*in situ* hybridization”, “phytoestrogens”, and “enteral nutrition” in 2000 to “fibrosis”, “women”, “complication”, “metformin”, “inflammation”, “chain fatty acid” in recent years.

### 3.8 Thematic evolution in endocrinology and metabolism research (2010–2023)

**Figure 9:**
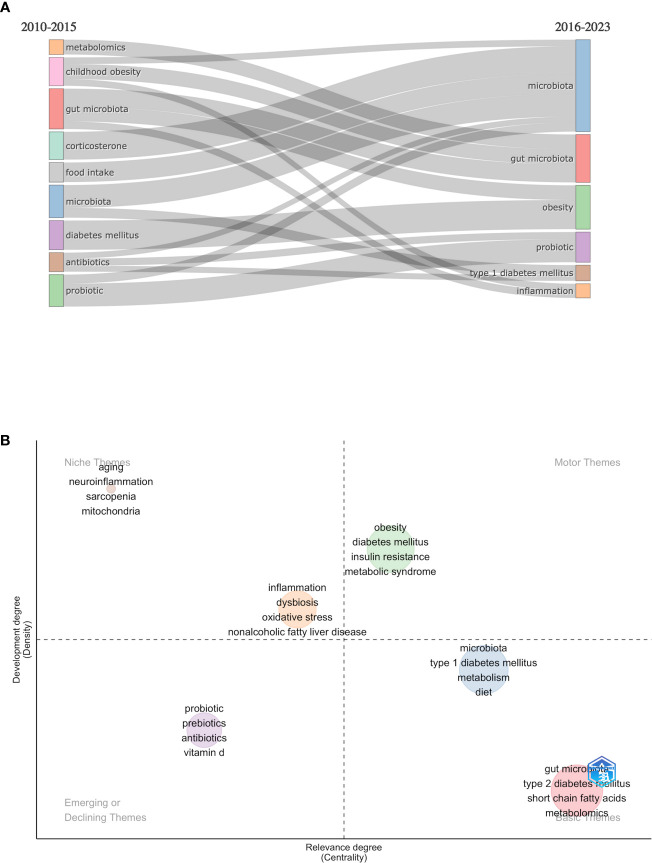
Thematic evolution and map of research themes from 2010 to 2023. **(A)** Transition of research themes from 2010-2015 to 2016-2023: The flow diagram illustrates the shifting focus of research themes over time. The left side represents the period from 2010-2015, and the right side depicts the period from 2016-2023, showing how certain themes have evolved or emerged as focal points of research. **(B)** Thematic map of 2016-2023: The thematic map categorizes research themes into four distinct areas based on their development density and centrality. Niche themes indicate specialized areas of research with growth potential. Motor themes represent the current and popular research direction of the research field. Basic themes show a sustained research interest. Emerging or Declining themes demonstrate newly emerging or declining topics and potential future significance.

Our investigation into the post-2010 period of scientific productivity in endocrinology and metabolism has bifurcated the time into two distinct intervals for a thematic evaluation. The thematic evolution analysis reveals a sustained and growing interest in “probiotics” and “microbiota” from 2010 to 2023. The first half of the period 2010-2015 saw a prominent focus on “childhood obesity”, “gut microbiota”, and “diabetes mellitus”. This period also saw significant interest in research into “probiotics” and “antibiotics”, as well as “metabolomics” (**Figure 9**).

The theme of “gut microbiota” continued its importance in this field by expanding to include themes such as “obesity” and “inflammation” in the 2016-2023 period. Additionally, there has been an increasing research interest in “type 1 diabetes” and “inflammation.” In particular, the focus has shifted from “childhood obesity” to a broader interest in “obesity” as a research theme. The enduring importance of the “probiotic” theme underlines the continued interest in therapeutic interventions in the field of gut microbiota.

### 3.9 Thematic distributions and trends

A detailed thematic map has been created to visually represent the density and centrality of various research themes in the endocrinology metabolism category from 2010 to 2023. This period was divided into two equal time slices. To analyze the current theme, the themes between 2016 and 2023 were assessed (**Figure 9**). The 2010-2015 time slice was uploaded as **Supplementary Material**. The map depicts the research landscape, capturing a snapshot of recent changes and emerging developments in the field.

On the left side of the map, “aging”, “neuroinflammation”, and “mitochondria” are identified as niche themes. These themes reflect targeted research areas exploring the intersections of endocrine-metabolic health with aging processes, neural inflammation, and cellular metabolism. Despite currently exhibiting lower centrality, their developmental density suggests these are budding fields with the potential to significantly influence future research directions. Other notable niche themes, such as “inflammation”, “dysbiosis”, “oxidative stress”, and “nonalcoholic fatty liver disease”, are positioned close to the center, suggesting a high likelihood of evolving into the motor or basic themes.

Themes “obesity”, “diabetes mellitus”, “insulin resistance”, and “metabolic syndrome” constitute the motor themes. These themes have a broad impact on the field of research and represent fundamental concepts relevant to many different studies.

The themes of “microbiota,” “type 1 diabetes,” “metabolism,” and “diet”, as well as “gut microbiota,” “type 2 diabetes mellitus,” “short-chain fatty acids,” and “metabolomics,” are situated within the basic themes category. This indicates that these themes form a general foundation for the research field and possess a broad scientific impact. They represent the core of the discussions and are supported by a considerable volume of research and citations.

Emerging themes such as “probiotics” and “prebiotics” are gaining interest, indicating a rise in the significance of gut health in systemic diseases. This trend reflects an expanded understanding of how modulation of the gut flora can influence metabolic pathways and overall health. Similarly, “vitamin D” and “antibiotics” have been noted as themes in the Emerging/Declining category.

## 4 Discussion

In this study, we aimed to explore the growing field of gut microbiota research within the context of endocrine and metabolism, employing advanced visual analysis software to dissect the academic discourse. This growth indicates a rapidly expanding area of scientific interest, with the potential for ongoing contributions through research publications. Furthermore, our analysis has revealed the prominent authors, seminal articles, and primary sources that influence the development of this evolving field.

Choosing the WoSCC as our database was instrumental in conducting a thorough and wide-ranging bibliometric study. The WoSCC, recognized for its extensive interdisciplinary scope, hosts over 170 million articles from more than 250 academic disciplines (Shi et al., 2022). Specifically, the database hosts a significant volume of literature pertinent to our study, with a considerable number of articles and cited references directly related to gut microbiota research within the realm of endocrinology and metabolism (Zhang et al., 2022; Li C. et al., 2023; Xue et al., 2023; Takeuchi et al., 2023). Given the database’s extensive usage and acclaim in scientometric research, it provides a solid basis for analyzing the trajectory of gut microbiota research in our field. We meticulously curated our dataset from this robust collection, ensuring a focus on high-quality, peer-reviewed journals well-regarded within the scientific community.

Our bibliometric analysis underscores highlighting a surge in publications post-2000 with a notable increase around 2008-2010. This trend underscores the field’s dynamic nature and its potential to contribute to understanding endocrine diseases, demonstrating its growing significance in the scientific community. The analysis recognizes the contributions of distinguished researchers like Cani PD and colleagues, whose work from 2007 to 2010 has profoundly influenced our current understanding of the microbiota’s role in metabolic health (Cani et al., 2007; Cani et al., 2008; Liu et al., 2021). Their pioneering studies on obesity, metabolic endotoxemia, and the immune system’s responses have established major milestones in the science of microbiota. They have exemplified the impact of changes in the composition of the gut microbiota, increased intestinal permeability, and metabolic endotoxemia characterized by increased circulating lipopolysaccharides (LPS), and subsequent inflammation on the development of metabolic diseases (Cani et al., 2007; Cani et al., 2008). These studies elucidate the complex interactions between gut microbiota and host health outcomes, guiding future research and a basis for developing microbiota-focused therapeutic strategies. Building upon this, targeted research has illuminated the roles of specific bacteria. Studies have demonstrated that *Lactobacillus reuteri* and *Akkermansia muciniphila* show a negative correlation with serum LPS levels and fasting plasma glucose levels (Shi et al., 2022; Li C. et al., 2023). This indicates these bacteria become a potential therapeutic target for reducing serum LPS levels and improving glucose metabolism.

Of the top 10 countries producing the highest number of articles, all except China and Iran are developed countries. This result may reflect the solid financial and institutional support behind it. In the dataset, Iran’s significant increase in the number of publications in the last 5 years is noteworthy. This points to the global importance of microbiota research in different geographical and economic contexts, indicating a diversifying research landscape. The rate of international collaboration was lower than the average in countries with a high number of articles, such as the United States and China. Efforts by these countries to enhance their international collaboration rates in this field could lead to faster and more effective growth in the field. Increasing international collaborations in microbiota and endocrinology metabolism research offers the potential for broader perspectives and faster advancements, tapping into diverse expertise and granting access to invaluable resources. This collaborative approach can catalyze innovation and accelerate breakthroughs in the field.

Our study utilized Bradford’s Law and h-index analysis to identify leading journals in this field. Bradford’s Law highlighted *Frontiers in Endocrinology*, which has seen a rapid increase in publications since its first relevant article in 2014. This suggests that authors increasingly prefer this journal. The h-index evaluation revealed *Cell Metabolism* as the most influential journal, with *Diabetes* and *Diabetologia* also emerging as key publications due to their significant contributions to diabetes and microbiota research. In the future, as the volume of research on diabetes, insulin resistance, and microbiota continues to grow, we may witness these two journals becoming core journals as well. This analysis underscores a dynamic and evolving field, with these journals playing pivotal roles in disseminating crucial findings.

The thematic analysis highlights the growing importance of obesity, diabetes, insulin resistance, and metabolic syndrome as motor themes and these topics have consistently led to advances in the field and attracted intense academic interest. The gut microbiota plays a critical role in energy harvest through the fermentation of indigestible carbohydrates, positioning it as a key regulator in host metabolism. Within this context, specific bacterial species such as *Akkermansia muciniphila* have been identified for their distinctive roles in modulating metabolic pathways, thereby presenting potential targets for therapeutic intervention. This bacterium, residing in the mucus layer, enhances the thickness and integrity of the mucosal layer and contributes to the prevention of inflammation caused by disturbances in gut permeability (Xue et al., 2023). Furthermore, studies have linked the presence of *Akkermansia muciniphila* with favorable impacts on glucose metabolism, suggesting a negative correlation with markers of insulin resistance and obesity, thereby offering potential therapeutic avenues for metabolic disorders (Shi et al., 2022). Moreover, research conducted by Takeuchi et al., has elucidated the role of gut microbiota components, notably *Alistipes indistinctus*, in metabolic regulation. This bacterium has been spotlighted for its capacity to enhance insulin sensitivity and modulate lipid profiles, potentially through its involvement in SCFA production and interaction with host metabolic pathways (Takeuchi et al., 2023).

Bacterial metabolites, particularly SCFAs have emerged as a basic theme in thematic analysis. The focus on SCFAs serves as an essential in understanding the intricate relationship between diet, microbiota composition, and host metabolism. SCFAs, produced by the fermentation of indigestible carbohydrates, underscore the local and peripheral endocrine effects mediated by the gut microbiota (Liu et al., 2021). These metabolites, including butyrate, play pivotal roles in maintaining intestinal barrier integrity, regulating immune responses, and influencing systemic health outcomes such as obesity and insulin sensitivity (Mayorga-Ramos et al., 2022). Notably, *Faecalibacterium prausnitzii*, a member of the Firmicutes phylum along with other butyrate-producing species such as *Clostridium butyricum* and *Clostridium kluyveri*, plays a crucial role in these processes (Parsaei et al., 2021). Over the last decade, SCFAs have attracted significant interest due to their effects on host immune responses and barrier functions. Generally, literature underscores the critical role of gut microbiota and SCFAs in human health and disease prevention, highlighting the need for further research to fully understand their mechanisms of action.

Specific niche areas of research promise to yield significant impacts, potentially shaping the future of the entire field. Themes such as inflammation, neuroinflammation, aging, and oxidative stress are earmarked as budding topics likely to command the focus of future research endeavors. Recent studies have highlighted the important role of the gut microbiota in modulating neuroinflammation, and the microbiota has been implicated as an important player in the Gut-Brain Axis (Megur et al., 2021; Solanki et al., 2023). Bacterial metabolites increase intestinal permeability and LPS are some of the mechanisms by which they can exacerbate systemic inflammation and contribute to neuroinflammation. Research in this specific area is likely to reveal detailed mechanisms of how the microbiota exerts its influence on the body, potentially offering deeper insights into the pathophysiology of endocrine disorders and overall health. Again, specific insights in this area could open new doors for understanding and treating diseases.

Another noteworthy observation in thematic analysis is the growing interest in probiotics and prebiotics, which indicates a shift towards exploring the modulation of the microbiota for the maintenance of overall health and the development of therapeutic approaches. The continued presence of antibiotics in the thematic map suggests a maturation within this area of research, potentially indicating that future studies may place less emphasis on this theme as the field evolves. Modulating the gut microbiota represents a promising approach for treating diseases associated with imbalances in the gut microbiota. Methods used for this modulation include prebiotics, probiotics, and fecal microbiota transplantation (FMT) (Huda et al., 2021; Mahboobi et al., 2018). Prebiotics, which are indigestible food components, particularly promote the growth of beneficial gut bacteria such as *Lactobacilli* and *Bifidobacteria*. Human studies have demonstrated that prebiotics have positive effects on metabolic and inflammatory markers, notably improving glycemia, especially with probiotics (Mahboobi et al., 2018; Bock et al., 2021). Probiotics, defined as live microorganisms that confer health benefits (e.g., *Bifidobacterium* and *Lactobacillus*), have been shown to reduce fasting blood sugar and HbA1c levels in patients with type 2 diabetes (Huda et al., 2021; Bock et al., 2021). FMT involves the transfer of fecal bacteria from healthy donors to patients and has been proven effective, especially in treating *Clostridium difficile* colitis (Spinner et al., 2020). Researchers are exploring FMT as a method for gut microbiota modulation based on these successes.

## 5 Limitations and future studies

This study exhibits some limitations inherent to bibliometric analysis. One notable limitation is the potential for issues of author name ambiguity to arise, especially when multiple authors share the same or similar names. Ambiguities stemming from identical author names can lead to inadvertent misattribution of publications and potentially undermine the accuracy of author-specific metrics. To mitigate this issue, among the most productive authors, we conducted a thorough analysis of publications authored by researchers who shared the same names. This deliberate approach was adopted to help alleviate the potential challenges posed by author name ambiguity. Furthermore, while the analysis was based on data from the WoS database, which is known for its comprehensiveness, it remains important to acknowledge the limitation of database specificity. While WoS is valuable for hosting high-quality and reputable journals, we must acknowledge that it does not cover every publication in the examined field. It’s quite comprehensive, but some valuable studies might be missed because they are not published in journals that meet WoS’s criteria. This could affect the breadth of our perspective and the depth of our bibliometric analysis.

Additionally, the approach the study focused on, specifically targeting articles indexed under “Endocrinology and Metabolism” in WoS, may inadvertently exclude relevant research published in multidisciplinary or alternative categories that retain relevance to the study’s topic. Moreover, the analysis is subject to language bias, as non-English articles that may contain valuable information are often underrepresented in databases such as WoS. These limitations should be taken into account when interpreting the findings of this bibliometric study.

For future research, we recommend broadening the bibliometric analysis by integrating additional databases such as PubMed and Scopus. This would enrich the bibliometric study, allowing for a wider scope of research trends and contributions to be explored. When feasible, combining data manually from multiple databases or utilizing sophisticated data processing methods can resolve limitations and provide a more robust and nuanced understanding.

## 6 Conclusion

In the ever-expanding field of academic medicine, our bibliometric analysis has illuminated the dynamic landscape of gut microbiota research in the context of endocrine metabolism. The surge in publications after 2010 underscores the growing scientific interest in this field and promises continued contributions through research publications. While international collaboration is promising, fostering more collaborations, especially in countries such as the United States, Japan, China, and Korea, will accelerate progress in the field. The leading journals in this domain include *Cell Metabolism*, which stands out in terms of citations, and *Frontiers in Endocrinology*, which leads in the number of publications. As the field matures, *Diabetes* and *Diabetologia* are poised to become the core journals. Distinguished authors like Cani PD, Bäckhed F, Nieuwdorp M, and Burcelin R have made indelible marks with their pioneering work in uncovering the profound effects of gut microbiota on endocrine and metabolism.

Our keyword and thematic analyses have consistently identified gut microbiota, obesity, diabetes, and inflammation as core research themes. Additionally, the emerging roles of the gut microbiota and probiotics and prebiotics as key players in health and disease prevention underscore the significance of this field. Our analysis paints a picture of an evolving and promising scientific landscape where international collaboration, influential journals, and forward-thinking researchers converge to unravel the complex interplay between gut microbiota and endocrinology. This landscape reveals that research in endocrinology and metabolism is increasingly focused on the connection between gut microbiota and diseases. It indicates a trend toward personalized medicine with microbiota modulation in this field. Future research should focus on integrating these findings into clinical practice, moving from lab-based studies to real-world patient care.

## Data availability statement

The raw data supporting the conclusions of this article will be made available by the authors, without undue reservation.

## Author contributions

DD: Conceptualization, Methodology, Visualization, Writing – original draft, Writing – review & editing. TÇ: Investigation, Software, Supervision, Writing – review & editing.

## Supplementary material

Supplementary Material 1 Thematic map of the 2010-2015 time slice.
