# Supplementary figures and images for: Research trends on the gut microbiota in endocrine metabolism: a thematic and bibliometric analysis
Source: Front Cell Infect Microbiol. 2024 Mar 22;14:1371727. doi: 10.3389/fcimb.2024.1371727 (PMC10995354; doi:10.3389/fcimb.2024.1371727)

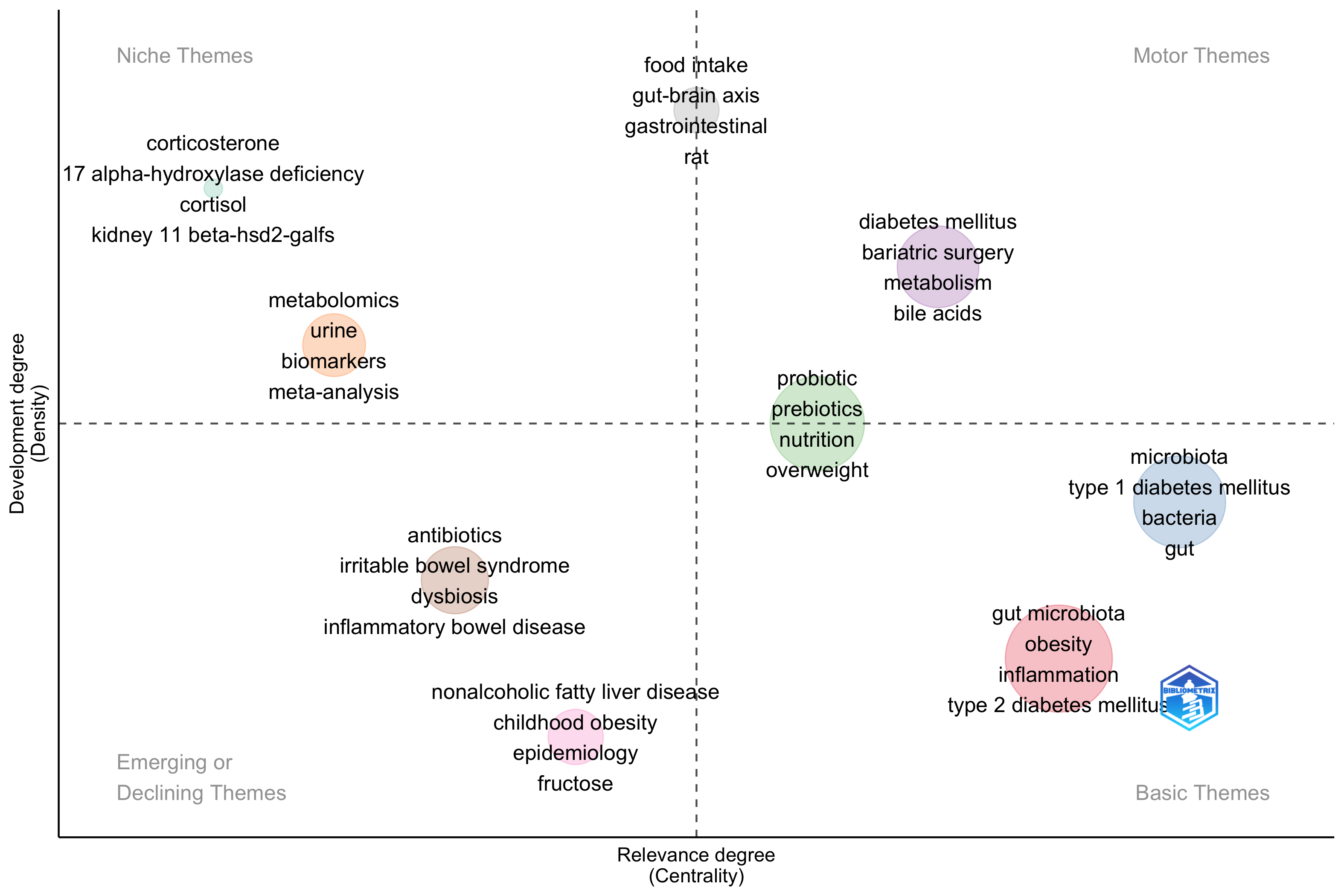

Supplement: Supplementary Material 1 — Thematic map of the 2010-2015 time slice. [file Image_1.png]
